# Heavy Metal Pollutions: State of the Art and Innovation in Phytoremediation

**DOI:** 10.3390/ijms20143412

**Published:** 2019-07-11

**Authors:** Giovanni DalCorso, Elisa Fasani, Anna Manara, Giovanna Visioli, Antonella Furini

**Affiliations:** 1 Department of Biotechnology, University of Verona, Strada Le Grazie 15, 37134 Verona, Italy; 2 Department of Chemistry, Life Sciences and Environmental Sustainability, University of Parma, Parco Area delle Scienze, 11/A, 43124 Parma, Italy

**Keywords:** phytoremediation, heavy metals, hyperaccumulation, plant genotype improvement, soil management

## Abstract

Mineral nutrition of plants greatly depends on both environmental conditions, particularly of soils, and the genetic background of the plant itself. Being sessile, plants adopted a range of strategies for sensing and responding to nutrient availability to optimize development and growth, as well as to protect their metabolisms from heavy metal toxicity. Such mechanisms, together with the soil environment, meaning the soil microorganisms and their interaction with plant roots, have been extensively studied with the goal of exploiting them to reclaim polluted lands; this approach, defined phytoremediation, will be the subject of this review. The main aspects and innovations in this field are considered, in particular with respect to the selection of efficient plant genotypes, the application of improved cultural strategies, and the symbiotic interaction with soil microorganisms, to manage heavy metal polluted soils.

## 1. Introduction

Like all living organisms, plants require chemical elements that are used as cofactors in biochemical reactions, as components of structural proteins and macromolecules, and as regulators of the electrochemical balance of cellular compartments [1]. Soil availability of nutrient elements fluctuate due to temperature, precipitation, soil type and pH, oxygen content, and the presence or absence of other inorganic and organic compounds. Being sessile organisms, plants developed adaptive and flexible strategies for sensing and responding to fluctuations in element availability to optimize growth, development, and reproduction under a dynamic range of environmental conditions. In addition, once taken up, elements must be allocated to different organs, cell types, and tissues through tight homeostasis mechanisms to ensure metal requirement, storage, and re-mobilization under different environmental conditions [2]. 

Heavy metals are naturally occurring elements, which are widely distributed in the Earth’s crust; they derive from rocks of volcanic, sedimentary or metamorphic origin, but in recent years, the prevalence of heavy metals in areas of agricultural and industrial activities has increased because of human activity [3]. A limited number of heavy metal ions are water soluble upon physiological conditions and thus bioavailable to plants and other living organisms, being either essential or potential risks for life [4]. Indeed, many heavy metals (mainly Fe, Zn, Cu, Ni, Co, and Mo), which are toxic when present in excess, are essential for plant and cellular biochemistry being involved in cell protection, gene regulation, and signal transduction and their absence (or deficiency) inhibits plant growth, reproduction, and tolerance to environmental stresses [5]. Other heavy metals such as Cd, Hg, Ag, Pb, and Cr are biologically non-essential and show toxicity even at low concentrations. The similarity of certain non-essential metals to essential ones allows the latter to enter plants replacing their essential homolog and interfering with biological functions. To minimize the unfavorable effects of non-essential heavy metals, while maintaining the uptake of essential elements, plants have evolved a homeostatic network that controls metal uptake, trafficking, storage, and detoxification. Although a basal metal tolerance is usual, to guarantee the correct concentration of essential metal nutrients in different cell types at different stages of plant development, plants have acquired complex mechanisms to avoid or overcome the harmfulness of heavy metal excess. In metal-rich soils, plants have evolved mechanisms to tolerate, within a certain limit, metal toxicity. Plants encountering heavy metals employ two main approaches: the most common strategy is metal exclusion, in which metal accumulation is limited to the belowground organs. Uptake and root-to-shoot transport are regulated to maintain low shoot content over a wide range of external concentration. On the opposite, plants can accumulate metals, and an extreme evolution of this capacity is well represented in metal (hyper) accumulators, which are able to accumulate heavy metals in their shoots keeping low concentrations in roots. This trait is associated with the enhanced ability to detoxify high metal levels in the aboveground tissues [6]. Both strategies are regulated by finely tuned homeostatic mechanisms to guarantee sufficient metal uptake, transport, accumulation, and detoxification.

## 2. Plant Mechanisms for Heavy Metal Tolerance and Detoxification

Relevant components of homeostatic networks underneath metal tolerance and detoxification include ion transporters, metallo-chaperons, and ligands that act in concert to ensure metal uptake, transport to different cell types and delivery inside cells. Membrane proteins are able to transport different metals across cellular membranes, playing a pivotal role in each influx-efflux step of the translocation from roots to shoots. The function of several transporters involved in import, trafficking, sequestration, and export of essential metals across the plasma membrane, tonoplast, or chloroplast envelope has been clarified [2,7,8]. Metal transporters have been classified into families according to sequence homology. For example, the ZIP family (ZRT-IRT-like proteins) is involved in several homeostatic processes including uptake and translocation from root to shoot [9,10]. The NRAMPs (naturally resistant associated macrophage proteins) comprises members such as: NRAMP1, which when in *A. thaliana* is localized in the plasma membrane, is involved in Fe transport, and also shows high-affinity Mn uptake from soil [11]; NRAMP3 and NRAMP4, which are localized in the tonoplast and are essential for exporting stored Fe from the vacuole during seed germination [12]. The HMA proteins (heavy metals P1B-type ATPases) contribute to pump cations out of the cytoplasm by ATP hydrolysis. HMA1 localizes in the chloroplast envelope and is possibly involved in plastid Zn detoxification under Zn excess [13]. Similarly, HMA3 is involved in the detoxification of Zn, Cd, Co, and Pb by regulating their sequestration into the vacuole, and HMA4, a plasma membrane transporter, plays a role in Zn efflux from the cytoplasm and xylem loading/unloading [14,15]. Another group of transporters that tightly regulate metal homeostasis ensuring the appropriate metal supply to tissues is represented by the CDF (cation diffusion facilitator) family whose members are involved in the translocation of metals towards internal compartments and extracellular space [7]. Among them, several MTPs (metal tolerance proteins) have been described in a variety of plant species. The best characterized is MTP1, which is a vacuolar Zn^2+^/H^+^ antiporter involved in Zn tolerance, which in case of Zn excess accumulates Zn into the vacuole [16]. 

In addition to metal trafficking, plant responses to heavy metal stress include a variety of mechanisms, ranging from changes in gene expression and methylation to metabolic and biochemical adjustments, with the final goal of scavenging toxic metal ions, and ameliorating stress symptoms and damages. The production of hormones such as ethylene, jasmonic acid, and abscisic acid is also induced, as well as molecules involved in chelation of metal ions, such as organic acid, specific amino acids, phytochelatins, and metallothioneins [17,18]. Proline and histidine induce tolerance by chelating ions within cells and xylem sap [19]. The induction of phytochelatins occurs because of high levels of different heavy metals although Cd seems to be the most effective stimulator [20]. As opposed to phytochelatins, which are produced enzymatically, metallothioneins are gene-encoded polypeptides that play a role in the homeostasis and sequestration of intracellular metal ions [21,22]. Chelating compounds contribute to heavy metal tolerance by removing toxic ions from sensitive sites through sequestration and subsequent vacuolar compartmentalization by tonoplast-localized transporters. When the above-mentioned strategies are insufficient to contain the damage, cells trigger the production of reactive oxygen species (ROS), which might potentially result in massive oxidative stress with cell homeostasis disruption, inhibition of most cellular processes, DNA damage and protein oxidation [23]. As a result, cells activate the ROS-scavenging machinery with the production of antioxidant compounds such as glutathione, flavonoids, and carotenoids as well as antioxidant enzymes including superoxide dismutases, catalases, and peroxidases.

## 3. Phytoremediation

As mentioned before, despite natural occurrence in soils, large quantities of heavy metals and metalloids have been dispersed into the environment by a variety of human activities including fertilizer use in agriculture, metal mining, and manufacturing by metallurgy, fossil fuel use, and military operations. Land contamination poses a serious risk to both human health and animal and plant biodiversity [24]. There are a variety of conventional approaches to reclaim contaminated sites that are usually based on physicochemical techniques, including soil washing, electric field application (electrokinetics), excavation and reburial of contaminated matrices, pumping and treating systems in case of polluted water. These approaches suffer from two main disadvantages, being expensive and frequently inefficient if pollutants are present at low concentrations. Moreover, harsh approaches cause significant changes to the physicochemical and biological characteristics of soils and landscapes. Ecological rehabilitation of contaminated sites may also be achieved by *phytoremediation*: an alternative in situ technology, which exploits plants and their rhizosphere to remove the contaminants or lower their bioavailability in soil and water with concurrent land revegetation [25]. 

### 3.1. Strategies for Phytoremediation

**Figure 1:**
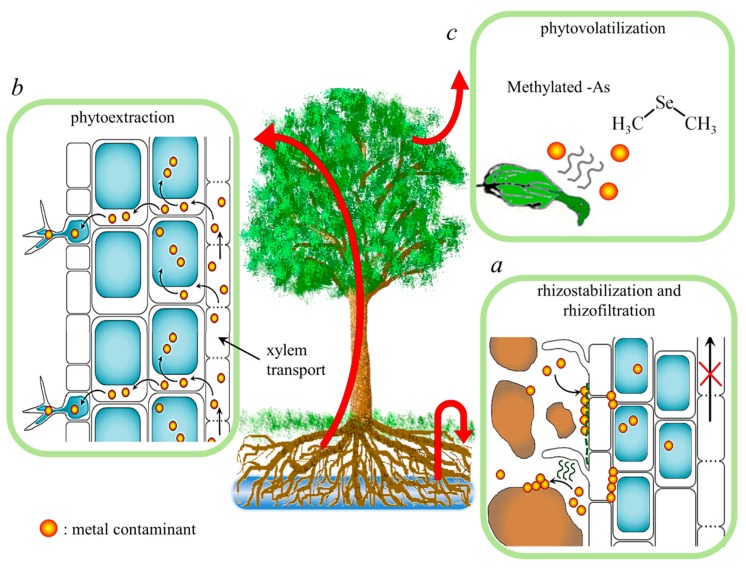
The main aspects of phytoremediation: the main steps involved in phytoremediation of heavy metals, which include (**a**) metal (yellow dots) adsorption on soil particles or cell walls (induced by rhizosphere metabolism) and compartmentalization of metals into root cell vacuoles (blue circles inside cells), preventing transport to the shoot; (**b**) metal accumulation in aerial organs (e.g., in vacuoles or trichomes) upon root-to-shoot xylem transport; (**c**) for particular metalloids (e.g., Se and As), leaf metabolism allows volatilization of the toxic compound.

Once placed in loco, plants deepen their root system into the contaminated soil matrix, establishing ecosystems with soil bacteria and fungi. Into this context, plants and the rhizosphere, i.e., soil and microorganisms associated to roots, employ mechanisms that altogether are responsible for the soil reclamation: phytodegradation, phytoextraction, phytovolatilization, phyto(rhizo)stabilization and phyto(rhizo)filtration (Figure 1). Such mechanisms are usually considered separately just for sake of clarity, even if they act in concert on the metal decontamination. In the following lines, each of these aspects will be highlighted individually, with the exclusion of phytodegradation, which is applicable to organic contaminants, rather than heavy metals, which are not degradable [26]. Plants acquire mineral elements from the soil primarily in the form of inorganic ions. The extended root system and its ability to absorb ionic compounds even at low concentrations make mineral absorption highly efficient. Obviously, heavy metals and metalloids can be absorbed by the plant root system, but since some of them, such as Cd and Pb, have no known biological function, it is likely that specific transporters do not exist. Indeed, toxic metals enter into the cells through cation transporters with a wide range of substrate specificity [18]. The ability of plants to take metals up and to accumulate them into the aboveground harvestable tissues is the rationale behind the second mechanism in phytoremediation, the so-called phytoextraction. Effective phytoextraction of metal-contaminated matrixes requires plants, which are characterized by a) efficient metal uptake and translocation to shoots; b) the ability to accumulate and tolerate high levels of metals; c) rapidly-growing and abundant shoots and deep root system. Some particular plants, commonly described as hyperaccumulator, show the ability to accumulate metals in aboveground tissues at very high concentration, without phytotoxic effects [27]. Unfortunately, most plant species displaying the hyperaccumulation trait are biennial or short-lived perennial herbs, shrubs, or small trees, characterized by a low biomass and a slow growth rate, which are major limitations for phytoextraction purposes [26]. Plants, which are suitable for effective phytoremediation, are therefore selected considering their tolerance to metal stress and biomass of aerial organs. For instance, it has been shown that *Populus* spp. and *Salix* spp. are able to accumulate relatively high foliar concentrations of metals such as Cd and Zn [28] and are often associated with metal-contaminated lands in northern Europe [29]. Interestingly, the evaluation on a time span of ca. 30 years of natural colonization on a contaminated site by different plant species, i.e., *Populus* ‘Robusta’, *Quercus robur*, *Fraxinus excelsior*, and *Acer pseudoplatanus*, has shown that the tree species determined a redistribution of metals in the soil profile, which was dependent on two main processes: the accumulation of metals in the leaves (an enhanced metal deposit into leaves contributes to an increased metal amount in the upper soil layer, upon seasonal leaf fall) and species-specific soil acidification (higher soil acidification by the root metabolism resulted in higher metal leaching from the upper soil layer with subsequent lower metal concentrations in such soil layer) [28]. Other than such biological aspects, successful phytoextraction is also guaranteed by lowering the time constraint of the process itself, especially evaluating the rate of metal pollutant removal and any eventual pollutant inputs [30]. 

When land contamination includes particular contaminants, such as Hg, As, and Se, plant metabolism is applicable to root absorption, translocation, and conversion of toxins into volatile compounds, which are released into the environment. Such a phenomenon, considered as a phytoremediation strategy, is called phytovolatilization. For instance, being chemically analog to sulfur, inorganic Se is converted to dimethyl selenide by plant enzymes involved in sulfur metabolism pathways, assimilation, and volatilization. Dimethyl selenide is dispersed into the air as a gas, which is significantly less toxic than inorganic Se [31]. Arsenic is another carcinogen and its contamination in soils is mainly due to natural sources and anthropogenic activities. Arsenite, formed in soils by the microbial activity, is readily taken up by plants, and some crops, e.g., rice, showed particular attitude to mobilize arsenite through the silicon uptake pathway, resulting in serious As poisoning to consumers [32]. The third metal that can be converted into volatile compounds is Hg, which is present in soils, waters and in the atmosphere. Leaf Hg content, mainly in the form of methyl-Hg, seems to derive almost entirely from leaf absorption by the atmosphere, since Hg transport through vascular tissues is very limited even considered that in particular paddy soils, chemical forms of water-soluble Hg can be promptly adsorbed and transferred to shoots, as observed in rice [33]. Experiments upon controlled conditions with wild-type *Brassica juncea* plants hydroponically treated with HgCl_2_ confirmed that upon root uptake, phytovolatilization of Hg is indeed happening in roots, rather than shoots due to the low root-to-shoot transport of the metal, and seems to occur via the metabolic activity of the root-associated algal and microbial community [34]. 

In soil, plant metabolism may contribute to the chemical stabilization of metal ions within the vadose zone, limiting leaching, mobility, bioavailability, and ultimately hazard. This process is known as phytostabilization and is accomplished by both metal ions absorption and accumulation in and onto roots, and by their precipitation in the rhizosphere zone due to binding by organic compounds and changes of metal oxidative state. Positively charged metal ions effectively bind to pectins in plant cell walls and to the negatively charged plasma membranes [35]. Plant species that accumulate heavy metals in their belowground parts are recognized as the most effective for phytostabilization, also known as rhizostabilization. Enhancement of phytostabilization processes is commonly obtained by coupling biological activity with soil amendment, in particular when dealing with heavily polluted soils. The utilization of inorganic soil additives, which include phosphate fertilizers, manganese, and iron oxides, clay and other minerals, and organic compounds, such as coal, compost and manure, aids plants by metal sorption and/or chemical alteration, as well as by beneficial effects on plant growth [36,37]. By reducing contaminants mobility and eventually the associated risks without necessarily removing them from the site, phytostabilization does not produce contaminated waste, such as harvested materials, which would need further treatments. 

The root metabolism of both terrestrial and aquatic plants can be also exploited to remediate polluted waters. This approach, named rhizofiltration, is used to absorb, concentrate, and precipitate metals from polluted water into plant biomass and its efficiency compares with currently employed water treatment technologies [38,39]. Early works demonstrated that a variety of aquatic plants, microorganisms, and seaweeds were able to biosorb metals and radionuclides dispersed in water (for a detailed description, refer to Section 3.2.2 in this review), but the lack of low-cost culturing, harvesting, and handling methods prevented full-scale testing [40]. It was in the early 1990s that the use of terrestrial plants grown hydroponically, able to achieve high above-water biomass and extensive root system to adsorb and absorb metals from contaminated liquids, got a foothold [41]. Mechanisms involved in rhizofiltration, also known as phytofiltration, mainly fall into three types characterized by different kinetics: a) sorption on the root surface, a quick component of metal removal, due to physical and chemical processes as chelation, ion exchange and specific adsorption (which do not include biological activity); b) processes that depend on plant metabolism, responsible for a slower metal removal from solutions and which rely on intracellular uptake, vacuolar deposition and eventually translocation to the shoots [42]; lastly, c) the slowest component of metal removal involves the release of root exudates which mediate metal precipitation from the solution in the form of insoluble compounds, as in case of phytostabilization. 

An interesting corollary of phytofiltration is the use of microalgae to treat municipal, industrial, agro-industrial, and livestock wastewaters. Microalgal bioremediation has been effective in the removal of toxic minerals such as, Br, Cd, Hg, and Pb, from effluents of food-processing plants and different agricultural wastes [43]. Moreover, algal biomass has found application for the passive biosorption of heavy metals in wastewater [43]. Recently, microalgae have drawn researchers’ attention due to their abilities in CO_2_ mitigation with environmentally beneficial outcomes, considering that CO_2_ is the largest contributor to the greenhouse effect [44]. Other than the above-mentioned features of microalgae, the main product of algal culture, i.e., the biomass, can be valorized for the production of biofuels, including biodiesel, biomethane, and biohydrogen [45], which is noteworthy in the context of the circular economy.

### 3.2. Advancement in the Field of Phytoremediation 

#### 3.2.1. Choosing the Best Plant Genotype 

**Figure 2:**
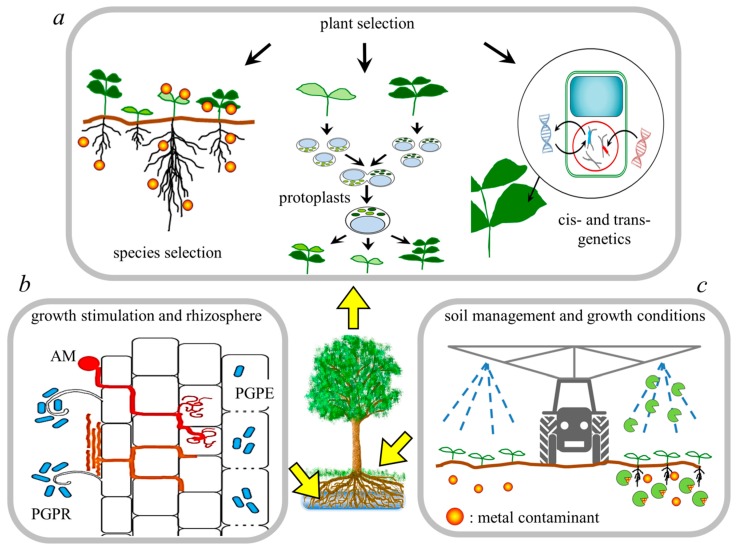
Main aspects of human intervention to enhance phytoremediation. Steps in which human activity can operate are, (**a**) the selection, through varietal choice, classical breeding (e.g., somatic hybridization), or transgenic approach, of the most useful plant species to be applied; (**b**) the enhancement of rhizosphere interconnections between plant growth promoting rhizo- and endophytic bacteria (PGPR and PGPE, respectively, blue dots), and mycorrhizal fungi (drawn in red -arbuscular mychorrizae, AM - and orange), exploiting ex-novo inoculum or the native microflora; (**c**) the management of the polluted site, in terms of both soil conditions and growth techniques, which can change metal availability, plant growth, and remediation effectiveness.

Enhancement of phytoremediation efficiency by increasing plant biomass, metal uptake or tolerance to metal toxicity is an important step in the development of new phytoremediation programs. The efficiency of phytoremediation can be improved through traditional approaches (such as plant breeding or hybridization and selection) or biotechnological techniques (i.e., the creation of engineered plants) that contribute to the development of plants with suitable phenotypes (Figure 2a).

Considering traditional approaches, improvement of plant phytoremediation efficiency was realized by the selection of wild non-edible ecotypes naturally growing in contaminated sites. Native plant species and populations, growing in metalliferous or contaminated sites, are able to cope with the high metal levels present in these soils; for this reason, they are much more resistant to these conditions than other plants and can be used for reclamation purposes [46]. Singh et al. [47] analyzed native plants growing on a site near the Uranium mine tailing ponds in Jaduguda and Turamdih, in the Jharkand State (eastern India), contaminated with heavy metals (Al, V, Ni, Cu, Zn, Fe, Co, Se, Mn) and radionuclides. Among the plants able to accumulate toxic metals and remediate the contaminated site, the As hyperaccumulator *Pteris vittata* was identified as the most versatile as it could accumulate Al, V, Ni, Co, Se, and U. Barrutia et al. [48] identified and characterized native plants spontaneously growing on soils from an abandoned Pb-Zn mine containing toxic levels of Cd, Pb, and Zn in the Basque Country (northern Spain). Among these, 31 species were able to accumulate and tolerate metals, including *Festuca rubra*, *Noccaea caerulescens*, *Jasione montana*, *Rumex acetosa*, and *Plantago lanceolata*. Shoots of *N. caerulescens* accumulated the highest Zn concentrations. Moreover, in vitro and greenhouse selections are suitable for the obtainment of heavy metal-tolerant plants, useful for soil remediation. *Daphne jasminea* and *Daphne tangutica* shoots were cultivated in vitro in the presence of different concentrations of Pb(NO_3_)_2_. In these conditions, *D. tangutica* accumulated high Pb concentrations, and chlorophyll and carotenoid biosynthesis were higher in comparison to *D. jasminea* [49].

To enhance the phytoremediation efficiency, genetic determinants of heavy metal accumulation and tolerance associated with wild hyperaccumulator species can be introduced by introgression into the genome of plants with significantly higher biomass [50]. For instance, *Brassica juncea* protoplasts were fused with *N. caerulescens* protoplasts to transfer the metal-resistant ability of *N. caerulescens* into *B. juncea* by somatic hybridizations. Hybrid plants showed the high Zn and Ni accumulation potential and tolerance derived from *N. caerulescens*, and the high biomass production specific of *B. juncea* [51].

Despite positive results obtained using classical breeding and genetic approaches, molecular engineering may be helpful to enhance plant phytoremediation potential and efficiency for the reclamation of polluted sites. Recombinant DNA technologies currently used for both nuclear and cytoplasmic genome transformation and the availability of genome sequences for different plant species allow the transfer of desirable determinants from hyperaccumulator species to sexually incompatible and high-biomass crops, suitable for in field phytoremediation. For instance, genetic engineering can be exploited to enhance metal tolerance and accumulation by the introduction of genes responsible for metal uptake, transport, accumulation, and detoxification, and for the response to oxidative stress, or to increase biomass production of hyperaccumulator plants [26]. Considering the reclamation of metal contaminated sites, good prospects come from the genetic engineering of high biomass species and trees, such as poplar. Eastern cottonwood trees (*Populus deltoides* Bartr. ex Marsh.) were genetically engineered by the introduction of the bacterial *merA* (mercuric ion reductase that reduces Hg^2+^ to the less toxic Hg^0^, which is volatized by plants) and *merB* (organomercury lyase that converts organic Hg to Hg^2+^) genes isolated and modified from *Escherichia coli* for the reclamation of mercury-contaminated sites. In vitro, *merA*/*merB* plants were more resistant to phenylmercuric acetate than wild-type controls and could detoxify organic Hg more efficiently [52]. Furthermore, genes that are currently widely used to improve plant phytoremediation potential are those that encode transporters of metal ions [26]. For example, Shim et al. [53] produced genetically engineered Bonghwa poplar (*Populus alba* x *P. tremula* var. *glandulosa*) lines expressing the yeast *ScYCF1* gene (*Saccharomyces cerevisiae -* yeast cadmium factor 1), which encodes a vacuolar transporter involved in toxic metal sequestration into the vacuole. When grown on a heavy metal contaminated soil from a mining site in South Korea, *ScYCF1*-expressing plants showed reduced Cd toxicity symptoms and accumulated much more Cd in comparison to wild plants. When plants were tested in the field on contaminated soil, dry weight and accumulation of Cd, Zn, and Pb in transgenic roots were significantly higher than in wild-types, demonstrating a potential utilization of these lines in long-term phytoextraction and phytostabilization of highly contaminated lands [53].

Among the genetic engineering tools, the clustered regularly interspaced short palindromic repeats (CRISPR)-Cas9 system is highly attractive to introduce a wide range of genes in different candidate organisms. CRISPR-Cas9 technology is a revolutionary and versatile gene-editing tool that can be used to enhance selected traits in plants targeting highly specific sequences of DNA [54]; for this reason, the technique could be used to transfer or modulate a desired set of genes in the plant genome to enhance the phytoremediation potential toward polluted soils and waters. The availability of genome sequences from model hypertolerant/hyperaccumulator species that may be considered for phytoremediation (such as the Cd, Ni, and Zn hyperaccumulator *N. caerulescens*, the Cd and Zn hyperaccumulator *Arabidopsis halleri* or the As hyperaccumulator *P. vittata*) and the improvement of bioinformatic tools have opened new opportunities for the use of CRISPR-Cas9 genome editing in the improvement of plant phytoremediation potential [55]. CRISPR-Cas9 technology could be used to introduce or modulate the expression of genes coding for metal transport proteins or involved in the synthesis of metal ligands [55]. Nowadays, despite the potential of this technique, its application in genome editing is still at an early stage in this field. However, there are few reports to date in which the CRISPR/Cas9 system has been successfully used for the reduction of metal content in plants. New rice lines knockout for the metal transporter gene *OsNRAMP5* were generated using the CRISPR/Cas9 system. These plants showed low Cd accumulation in shoots, roots, and in grains, upon hydroponic culture and in Cd-contaminated paddy field trials, maintaining biomass similar to wild-type [56]. This work provides a good example of the potential of the CRISPR/Cas9 system for the development of plants with a modified heavy metal content that could be used to achieve sustainable environmental cleanup via phytoremediation. In addition, phytoremediation could also benefit from the plant association with plant growth-promoting rhizobacteria [PGPR] (see following Section 3.2.3), where the CRISPR technology could be used to create more competent bacterial strains [55]. The application of the CRISPR-Cas9 technology to plants and PGPR, therefore, could be used to increase biomass yield and/or heavy metal tolerance, accumulation and detoxification, and thus to enhance their potential for the application in phytoremediation programs.

#### 3.2.2. Changing the Growth Conditions

In addition to the plant genotype used, cultural strategies have an enormous impact on phytoremediation efficiency (Figure 2c). Several phytotypologies, i.e., planting strategies, can be suitable to treat different pollutants taking into account the characteristics of the polluted matrixes [57]. Among the systems for the decontamination of liquid polluted matrixes, such as sewages, landfill leachates or storm water, constructed wetlands have been extensively applied. This strategy relies on floating and/or rooted hydrophytes and the associated microbiota to detain and remove pollutants mainly by rhizofiltration, although other remediation processes such as phytostabilization and extraction also occur. Free-floating macrophytes (e.g., *Lemna* spp., *Eichhornia crassipes,* and others) have been demonstrated to be excellent metal accumulators [58]; however, the rapid growth and invasiveness of many of these species are a double-edged sword and raise controversies regarding their application for phytoremediation [59]. For this reason, rooted plant species have found a wider application. Rooted hydrophytes (e.g., *Phragmites* spp., *Thypha* spp., *Cyperus alternifolius*, and others) and flood-tolerant species (e.g., *Chrysopogon zizanioides*) have been employed with excellent results for the construction of surface- and subsurface-flow wetlands (reviewed by [60,61]), as well as floating bed systems [62,63]. In constructed wetlands, the composition of growth beds plays a pivotal role. Almost complete removal of metal pollutants has been achieved by using porous materials such as crushed sea shell grits [64], stratified pomice and loamy soil [65], and zeolite [66], whereas composted green waste and gravel limit the remediation performance [64,66]. However, it is important to notice that this fundamental role of growth bed materials is imputable to their different absorption capacities. Indeed, evidence show that, in constructed wetlands, metal pollutants are partially retained by the medium depending on its properties, opening the question of growth bed disposal after the treatment [60].

Another strategy proposed for liquid waste treatment and disposal is that of land treatment, i.e., the application of polluted wastewater as irrigation for plant cultures. Although this system has been tested on different scales with some success [67,68], serious issues remain regarding its influence on soil characteristics, leaching in groundwater and overall impact on the environment [69,70]. As for polluted soils, the application of plant covers has been widely used for inorganic contaminants and relies on a variety of phytoremediation mechanisms, including phytoextraction and phytostabilization (refer to Section 3.1). Indeed, the plant covers effectively prevent pollutant dispersion and leaching in the groundwater, in addition to playing a more active role by removing it through uptake and detoxification [71]. Since metal-polluted soils often offer prohibitive growth conditions due to low nutrient content in addition to high metal levels, phytoremediation can be aided by good soil management practices to enhance global soil quality. In particular, the application of organic amendments, as for example manure or waste compost, has a positive effect on plant growth in phytoremediation [71,72]. Moreover, amendments can alter metal speciation, solubility, and bioavailability by altering water holding capacity, pH, and redox status of the soil [71], influencing the predominant phytoremediation strategy and the efficiency of the system. For example, cow manure, sewage sludge, and forest litter have been reported to enhance As extractability in As-polluted soils, favoring phytoextraction by ryegrass [73]. On the contrary, the application of sewage sludge and municipal waste compost reduced Cu, Zn, and Pb mobility in acidic metal-contaminated soil, leading to a more phytostabilization-oriented strategy [74].

Another widely considered practice to alter metal availability in polluted soils is the chelate-induced phytoextraction. The synthetic chelating agent ethylenediaminetetraacetic acid (EDTA) has proven highly efficient in metal mobilization [75], but its poor biodegradability causes concern due to its persistence in the environment, possible metal leaching and negative effects on soil properties and microbial communities [76]. As an alternative, biodegradable chelating agents as ethylenediaminedisuccinic acid (EDDS) and nitrilotriacetic acid (NTA), as well as organic acids, have been employed in several studies, enhancing phytoextraction efficiency [77,78,79].

Another supplementation that has been considered is that of exogenous phytohormones, with the aim of enhancing plant biomass, fitness and stress tolerance, thus improving phytoremediation efficiency. Cytokinins were demonstrated to produce generally positive effects in terms of plant growth and phytoextraction capacity [80], whereas conflicting results were achieved using auxins [81,82]. The application of gibberellins or some commercial growth regulator mixtures reduced metal accumulation in plant tissues, but the increased plant biomass brought still to a general enhancement of metal extraction per plant [83]. Moreover, the combined application of phytohormones with other treatments, such as nitrogen fertilization and chelating agents, produced a synergistic action resulting in a significant increase in metal phytoextraction [83].

In recent years, a novel strategy has been proposed to overcome some of the flaws of traditional phytoremediation, namely the long time and limited treatment depths. This technique, named electro kinetic-enhanced phytoremediation, relies on the combined application of plants and a physicochemical treatment, i.e., low-intensity electric fields, to the metal-polluted soil, favoring metal mobilization and bioavailability [84]. The application of different electrode materials and distribution, and different types of electric field have been considered with variable results [84]; however, a significant increase in Pb, As, and Cs phytoextraction has been achieved by DC electric field applied with inert low-cost graphite electrodes, due to the alteration of soil pH and metal solubility resulting from the electric field [85]. Interestingly, the application of a solar cell-powered electric field has been tested in a real-scale field trial for the phytoremediation of a metal polluted electronic waste recycling center by *Eucalyptus globulus*: the application of the electric field resulted in increased plant growth and metal accumulation. Moreover, although traditional power supply systems are more efficient in metal mobilization and containment, solar cells make this strategy significantly more sustainable on the economical level [86].

Finally, *nanoparticles* have also been considered for their possible use in assisting phytoremediation. Different types of nanomaterials have been applied for the decontamination of metal-polluted substrates thanks to their absorption capacity or redox catalytic activity [87]. In combination with plants, nanoparticles can be employed to improve the effectiveness of phytostabilization by absorbing metal ions [88], or phytoextraction by improving plant fitness and stress tolerance and increasing metal bioavailability [89,90].

#### 3.2.3. Enhancing the Plant-Microorganism Interactions

In recent years, researchers have focused their attention on the interactions between plant and metal resistant soil microorganisms, in particular, those colonizing roots (i.e., the rhizobiome) [91]. The synergism between plant roots and microorganisms can implement the remediation process by enhancing phytostabilization, as in the case of arbuscular mycorrhizae (AM) fungi, and phytoextraction employing plant growth promoting rhizo- and endo-bacteria [91] (Figure 2b). AM fungi establish mutual symbiosis with higher plants, improving mineral nutrition. Thus, AM contributes to plant growth in heavy metal contaminated sites by increasing plant access to nutrients such as P, by improving soil texture through the stable aggregation of soil particles and by binding heavy metals into roots restricting their translocation to shoot tissues. In that respect, AM fungi have been reported to reduce metal uptake and distribution in sunflower plants [92,93]. Therefore, AM fungi promote phytostabilization of heavy metals, accelerating the revegetation of severely degraded lands, such as coalmines or waste sites [94].

On the other hand, plant growth promoting rhizobacteria and endophytes (PGPR and PGPE, respectively) are able to increase the phytoremediation competence of plants by promoting their growth and health even under hazardous levels of heavy metals, by means of traits, such as organic acid production, secretion of siderophores, indole-3-acetic acid (IAA) production and 1-aminocyclopropane-1-carboxylate (ACC) deaminase activity. In most metalliferous soils, metals are strongly bound to soil particles, being not promptly available for plant uptake. Various PGPR and PGPE (e.g., *Arthrobacter*, *Microbacterium*, *Bacillus*, *Kocuria* and *Pseudomonas* spp.) can solubilize water-insoluble Zn, Ni, and Cu by local soil acidification through the secretion of protons and/or organic anions (e.g., acetate, lactate, oxalate, tartrate, succinate, citrate, gluconate, ketogluconate, and glycolate) [95]. Moreover, metal bioavailability in soils can be further increased by inoculating PGPR able to secrete biosurfactants, which can aid in metal ion release from soil particles [96]. Under iron-limiting conditions, PGPR secrete low molecular weight siderophores, which are iron chelators with an exceptionally strong affinity for ferric iron (Fe^3+^), enhancing its availability to both microorganisms and, indirectly, plants [97]. Siderophores are able to chelate several other metal species, such as Mg, Mn, Cr(III), Cd, Zn, Cu, Ni, As, and Pb with variable affinity. For instance, *B. juncea* plants inoculated with the mutant SD1 of the phosphate-solubilizing *Enterobacter* sp. NBRI K28, characterized by an enhanced siderophore production, showed increased biomass and phytoextraction of Ni, Zn, and Cr [98]. In addition to altering metal availability, a great majority of root-associated PGPR also produces the main bacterial auxin IAA, which promotes plant growth, stimulating root cell proliferation, lateral root initiation and overproduction of root hairs. Generally, bacterial IAA facilitates the adaptation of host plants in metal-contaminated sites by triggering physiological changes in plant cell metabolism under metal stress and helping plants to withstand high concentrations of heavy metals [99]. Several PGPR and PGPE are also able to synthesize the enzyme ACC deaminase, which degrades ACC (an immediate precursor of plant ethylene) into 2-oxobutanoate and ammonia, hence inhibiting ethylene production in plants, which is usually induced by heavy metal stress. It has been demonstrated that inoculation with ACC deaminase-producing PGPR resulted in extensive root proliferation in hyperaccumulator plants and efficient phytoremediation in metal-polluted soils [100]. From a technological point of view, microorganisms, which can be exploited for soil remediation or phytoextraction technologies, are usually members of complex metal-tolerant populations associated with tolerant and/or hyperaccumulator plant species growing in metalliferous soils. In some cases, PGPR and PGPE originally isolated from hyperaccumulator plants have been shown to promote growth and phytoextraction of diverse plant species grown in single and multiple metal-contaminated soils [101,102]. However, the impact of PGPR and AM fungi on different plants varies depending on the plant and microbial species and soil types. Several authors have tested PGPR and PGPE as bio-inoculum to remove different heavy metals from soils [103,104,105]. Zn-mobilizing bacteria, isolated from serpentine soils, promoted Zn, Cu, and Ni accumulation in *Ricinus communis* [106], while *Rahnella* sp. JN6, isolated from *Polygonum pubescens,* can promote growth and Cd, Pb, and Zn uptake in *B. napus* [107]. A bacterial consortium, isolated from the rhizosphere of the pseudometallophyte *Betula celtiberica* growing in an As-polluted site, enhanced As accumulation in leaves and roots, whereas the rhizobacterium *Ensifer adhaerens* strain 91R mainly promoted plant growth upon laboratory conditions [108]. Moreover, field experimentation showed that additional factors, such as soil As content and pH, influenced As uptake in the plant, attesting the relevance of field conditions in the success of phytoextraction strategies [108]. As for a phytostabilization-oriented strategy, AM fungi associated to the metallophyte non-accumulator *Viola calaminaria* inhabiting Zn- and Pb-rich soils were shown to improve maize growth in a polluted soil reducing heavy metal concentrations in plant tissues [109,110].

Nevertheless, it must be considered that the details of the interaction between plant roots and root-associated microorganisms are still rather unknown. Moreover, the rhizosphere is an extremely complex and still poorly characterized community: roughly, 99% of soil microbial taxa are yet to be cultured and can only be investigated using culture-independent methods [111]. At this purpose, approaches with *Omics* technologies, based on DNA, RNA, and protein sequencing have advanced our understanding of plant and microbial responses to pollutants and of plant–microbe interactions. For instance, high-throughput sequencing of bacterial 16S rDNA allows defining the composition of the microbial community and how heavy metals drive the selection toward microorganisms, which are more suitable for phytoremediation purposes [92]. In addition, transcriptomic and proteomic studies on rhizosphere communities in contaminated soils are instrumental to predict valuable microbial functions directly [112,113]. The integration of these strategies allows creating a complete picture of how cohabiting and symbiotic biological communities interact to adapt to metal stress and could enhance phytoremediation [114]. Eventually, these data need to be combined with high-throughput isolation and screening for key microbial characteristics such as growth rate, to target microbes that are perhaps not naturally dominant but have valuable traits for their application in phytoremediation [114].

Despite all the research in the field of plant-microorganism interaction, applications of PGPR and mycorrhizal consortia in assisted phytoremediation in contaminated soils are still scarce and the performance of these microorganisms under natural conditions needs to be more deeply investigated. A particular consideration is the biosafety linked to the release of non-autochthonous bacterial strains. In addition, even though such strains might be superior in terms of metal resistance and mobilization effectiveness, the competition with the native microbial population can reduce the efficacy of the inoculated strains. Despite these concerns, co-inoculation with PGPR and mycorrhizal consortia might partially mimic the natural conditions of contaminated soils, in which multiple microorganism interactions occur, helping plants to cope with the toxic effects of heavy metals. Co-inoculation can also improve the phytostabilization or phytoextraction efficiency for various metals at the same time, which indicates the possibility of exporting the technology to multi-metal contaminated sites [67,105].

## 4. Conclusions

Summarizing, plants and associated microorganisms surely are of great interest for their potential application in polluted soil reclamation. A variety of options are available when considering a phytoremediation approach, including the utilization of wild plant-microorganisms associations, or the implementation by applying particular planting and culturing techniques. Researchers can develop the best suitable plant lines or microorganisms to be exploited, as well as the best fertilizers or soil conditioners. Interestingly, attention moved also on the fate of contaminated biomass, particularly when dealing with approaches of phytoextraction. Indeed, recent research is aimed to valorize metal-rich biomass rather than simply dispose of it, coupling land reclamation with non-food products (e.g., timber) and energy production, a concept known as integrated phytoremediation. In this view, harvested plant biomass has a substantial calorific value in terms of renewable energy production. Therefore, long-term operations of planting, maintaining the phytoremediation site (otherwise unsuitable for remunerative and productive uses) and fruitfully converting harvested biomass, are grouped into the new idea of phyto-management, in which the major goal is mitigating environmental risk and making contaminated lands economically valuable [26,30].
